# Plasmapause surface wave oscillates the magnetosphere and diffuse aurora

**DOI:** 10.1038/s41467-020-15506-3

**Published:** 2020-04-03

**Authors:** Fei He, Rui-Long Guo, William R. Dunn, Zhong-Hua Yao, Hua-Sen Zhang, Yi-Xin Hao, Quan-Qi Shi, Zhao-Jin Rong, Jiang Liu, An-Min Tian, Xiao-Xin Zhang, Yong Wei, Yong-Liang Zhang, Qiu-Gang Zong, Zu-Yin Pu, Wei-Xing Wan

**Affiliations:** 1 Key Laboratory of Earth and Planetary Physics, Institute of Geology and Geophysics, Chinese Academy of Sciences, Beijing, 100029 China; 2 Innovation Academy of Earth Science, Chinese Academy of Sciences, Beijing, 100029 China; 3 Laboratoire de Physique Atmosphérique et Planétaire, STAR Institute, Université de Liège, Liège, B-4000 Belgium; 4 Mullard Space Science Laboratory, Department of Space and Climate Physics, University College London, Holmbury St Mary, Dorking, RH5 6NT UK; 5 Institute of Applied Physics and Computational Mathematics, Beijing, 100088 China; 6 Institute of Space Physics and Applied Technology, Peking University, Beijing, 100871 China; 7 School of Space Science and Physics, Shandong University, Weihai, 264209 China; 8 Institute of Geophysics and Planetary Physics, University of California, Los Angeles, CA 90095 USA; 9 Key Laboratory of Space Weather, National Center for Space Weather, China Meteorological Administration, Beijing, 100081 China; 10 Johns Hopkins University Applied Physics Laboratory, Laurel, MD 20723 USA

**Keywords:** Aurora, Magnetospheric physics

## Abstract

Energy circulation in geospace lies at the heart of space weather research. In the inner magnetosphere, the steep plasmapause boundary separates the cold dense plasmasphere, which corotates with the planet, from the hot ring current/plasma sheet outside. Theoretical studies suggested that plasmapause surface waves related to the sharp inhomogeneity exist and act as a source of geomagnetic pulsations, but direct evidence of the waves and their role in magnetospheric dynamics have not yet been detected. Here, we show direct observations of a plasmapause surface wave and its impacts during a geomagnetic storm using multi-satellite and ground-based measurements. The wave oscillates the plasmapause in the afternoon-dusk sector, triggers sawtooth auroral displays, and drives outward-propagating ultra-low frequency waves. We also show that the surface-wave-driven sawtooth auroras occurred in more than 90% of geomagnetic storms during 2014–2018, indicating that they are a systematic and crucial process in driving space energy dissipation.

## Abstract (web summary)

Theoretical studies suggested that plasmapause surface waves related to the sharp inhomogeneity exist and act as a source of geomagnetic pulsations. Here, the authors show direct observations of a plasmapause surface wave and its impacts during a geomagnetic storm using multi-satellite and ground-based observations.

## Introduction

The solar wind and embedded interplanetary magnetic field (IMF) play a crucial role in driving terrestrial magnetospheric energy dissipation. When the IMF has a southward component, the mass and energy in solar winds enter into and convect in the magnetosphere and are eventually released into the ionosphere and upper atmosphere, generating spectacular auroras in the Earth’s polar regions^1,2^. A large part of the electromagnetic energy is carried by ultra-low frequency (ULF; ~0.1 mHz to 10 Hz) waves that propagate throughout the system and couple different regions together. The ULF waves play important roles in generating quasi-periodic geomagnetic perturbations^3^ and energizing energetic particles in the Earth’s radiation belts^4,5^. It is commonly accepted that ULF waves can be driven externally by solar wind perturbations^6^ and magnetopause surface waves^7–9^, or internally by, for instance, plasma instabilities in the nightside magnetosphere^10^. Many auroral activities are related to the ULF waves in the magnetosphere, such as the substorm expansion phase onset^11^, the auroral arcs^12^, and the fluctuations of auroral intensity^13,14^. The ULF waves generated by the magnetopause surface wave propagate radially inwards^15^ and penetrate deep inside the inner magnetosphere during geomagnetic storms^16^, while it is unknown whether the reverse process can occur.

The inner part of the Earth’s magnetosphere, known as the plasmasphere, is full of cold (~1 eV) and dense plasmas that corotate with the planet^17^. The outer boundary of the plasmasphere is called the plasmapause, which separates two types of plasmas contents characterized by different temperatures and densities^18,19^. Energetic particles outside the plasmapause can precipitate into the middle- and high-latitude ionosphere along magnetic field lines and generate spectacular auroras in both hemispheres^20^. Under quiescent conditions, a longitudinally smooth equatorward boundary of the diffuse aurora is usually expected, corresponding to an azimuthally flat plasmapause boundary, which separates the hot ring current/inner plasma sheet from the cold plasmasphere^21^. During geomagnetic storms, especially in the main phase, the enhanced solar-wind-induced convection electric field can penetrate deep into the inner magnetosphere and erodes the outer part of the corotating flow to form a sharp plasmapause in radial direction^22,23^. On the basis of magnetohydrodynamic (MHD) theory, Chen and Hasegawa^24^ predicted that impulses acting on the sharp plasmapause boundary may result in a discrete eigenmode, i.e., a plasmapause surface wave (PSW), at the plasmapause surface. Nevertheless, direct evidence of the PSW has not yet been found from in situ measurements and the function of PSW on magnetospheric dynamics remains unclear.

Despite the fact that the diffuse auroral boundary is generally smooth, spectacular sawtooth-shaped large-scale undulations along the equatorward edge of the diffuse aurora (hereafter shortened to ‘sawtooth aurora’ (SA)) are observed in the afternoon-to-evening sector during geomagnetically disturbed periods^25–29^. Several plasma instability mechanisms^25,28,29^ have been proposed to interpret the origin of SAs, but a conclusion is far from imminent due to the lack of conjugated observations of the SAs and their magnetospheric source regions. Since the SA and the plasmapause are located at similar *L*-shells and possibly linked by the same magnetic flux tubes, a physical connection between PSW and SA is naturally expected and yet to be investigated.

Here we present a direct observational evidence of PSW using conjugated satellite and ground observations and demonstrate that it is a systematic ULF wave driver in the magnetosphere. We show that the SA on the equatorward edge of diffuse aurora generated by the PSW occur in more than 90% of geomagnetic storms, indicating that PSW is a systematic consequence of geomagnetic storms and has crucial impacts on energy dissipation in the ionosphere-magnetosphere coupling system.

## Results

### Evidence of PSW

**Fig. 1:**
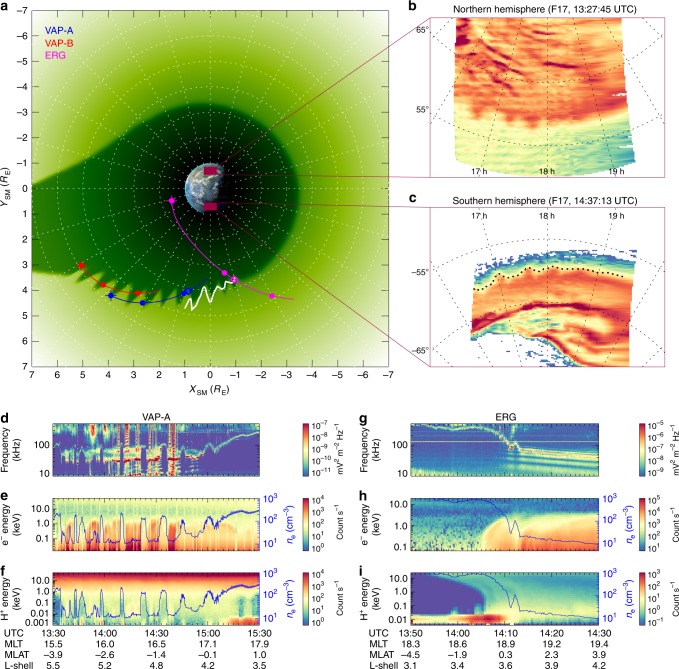
Coordinated observations of PSW and associated SA on 16 July 2017. **a** Schematic diagram showing the geometry of the plasmasphere (deep green area), the auroral boundary (white thick curve), and spacecraft trajectories (VAP-A, VAP-B, and ERG spacecraft shown in blue, red, and pink, respectively) on the equatorial plane in the solar magnetic (SM) coordinate system with the Sun to the left (see the Methods section, coordinate system). The shape of the plasmapause is calculated from an empirical plasmaspheric model^37^. The clear sawtooth-shaped plasmapause structure visible in the afternoon-dusk sector indicates the PSW, which is manually added based on the wavelength of the PSW calculated in the main text. The closed circles in the spacecraft trajectories indicate temporal intervals of 1 h beginning at 13:00 UTC (indicated by the leftmost circles overlaid with crosses). Both VAP-A and VAP-B spacecraft were located in the southern hemisphere while ERG spacecraft crossed the magnetic equator at ~14:10 UTC. Open diamonds represent the plasmapause crossings by the spacecraft. Note that VAP-A and VAP-B crossed the plasmapause region roughly azimuthally while ERG crossed almost radially. **b** SA observed by DMSP F17 in the Northern Hemisphere (NH) at 13:27:45 UTC. **c** SA observed by DMSP F17 in the SH at 14:37:13 UTC. The dashed grid lines in **b**, **c** denote the altitude-adjusted corrected geomagnetic (AACGM)^38^ latitudes and magnetic local time (MLT). The thick dotted line in **c** represents the boundary of the SA, which is projected onto the SM equatorial plane using the Tsyganenko 96 magnetic field model^36^ and denoted by the white curve at the duskside plasmapause in (**a**). **d** Spectrogram of the electric field from the high frequency receiver of the EMFISIS instrument suite onboard VAP-A. The next two panels show the energy spectrograms of electrons **e** and protons **f** measured by the HOPE mass spectrometer onboard VAP-A, respectively. The plasmaspheric electron density (blue curve) is overlaid on (**e**, **f)**. **g**–**i** The same as those of **d**–**f** but for the ERG spacecraft. Details of the instruments and data availability can be found in Methods (see the Methods section, data usage).

Figure 1a shows the satellite locations from 13:00 to 15:30 UT and the schematic plasmaspheric configuration on 16 July 2017 during the main phase of a geomagnetic storm under the southward IMF condition (Supplementary Fig. 1). The Van Allen Probes (VAP) A spacecraft^30,31^ (blue curve in Fig. 1a) observed alternating distributions of the cold and hot plasmas in the afternoon-to-dusk sector (Fig. 1d–f). Clear large-amplitude oscillations at the frequency of the upper hybrid resonance in the spectrogram of electric field (Fig. 1d) indicate that the plasmaspheric electron density (PED) (see the Methods section, calculation of PED) was oscillating along the spacecraft trajectory. A comparison between the electron density and the spectra of electrons (Fig. 1e) and protons (Fig. 1f) further demonstrates that the hot plasmas periodically intruded into the cold plasma regions. Similar distributions were observed by the VAP-B spacecraft (red curve in Fig. 1a) at the same time (Supplementary Fig. 2). The Exploration of energization and Radiation in Geospace (ERG, also called Arase) spacecraft^32–34^ (pink curve in Fig. 1a) also observed similar plasmas distributions in the dusk sector (Fig. 1g–i). These data reveal that these spacecraft were passing over the plasmapause several times, implying a sawtooth-shaped plasmapause undulation, as illustrated in Fig. 1a. During this period, the Defense Meteorological Satellite Program (DMSP) F17 satellite^35^ observed SA at the dusk side in both hemispheres (Fig. 1b, c). The SA boundary (thick dotted line in Fig. 1c) is mapped to the sawtooth-shaped plasmapause on the equatorial plane (white line in Fig. 1a) along the modeled magnetic field lines^36^. Clearly, the sawtooth-shaped plasmapause structure is the manifestation of the PSW that was propagating sunward/westward, as will be detailed below.

### Sunward/westward propagating PSW

**Fig. 2:**
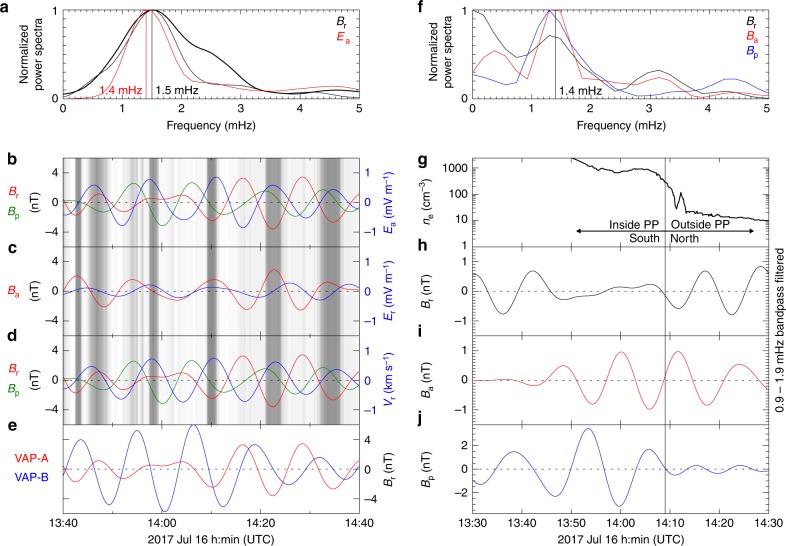
Spectral analysis of the magnetic field perturbations measured by VAP and ERG. **a** Normalized power spectra of the perturbations of the magnetic field radial component of the magnetic field *B*_r_ (black, thin for VAP-A and thick for VAP-B) and the electric field azimuthal component *E*_a_ (red) measured by VAP-A. **b** Bandpass filtered (1.5 ± 0.5 mHz) perturbations of *B*_r_ (red), field-aligned magnetic field *B*_p_ (green) and *E*_a_ (blue). **c** Bandpass filtered (1.5 ± 0.5 mHz) perturbations of the azimuthal magnetic field *B*_a_ (red) and the radial electric field *E*_r_ (blue). **d** Bandpass filtered (1.5 ± 0.5 mHz) perturbations of *B*_r_ (red), *B*_p_ (green) and radial ion velocity *V*_r_ (blue) (see the Methods section, data usage). The background in **b**–**d** represents electron density with dark color (colorless) for high (low) density inside (outside) the plasmasphere. **e** Bandpass filtered (1.5 ± 0.5 mHz) perturbations of *B*_r_ measured by VAP-A (red) and VAP-B (blue). **f** Normalized power spectra of the perturbations of *B*_r_ (black), *B*_a_ (red), and *B*_p_ (blue) measured by ERG. The vertical line indicates a peak at 1.4 mHz. **g** Radial profile of the plasmaspheric electron density. **h**–**j** Bandpass filtered (1.4 ± 0.5 mHz) perturbations of *B*_r_, *B*_a_, and *B*_p_, respectively between 13:30 and 14:30 UTC. The thick vertical line indicates crossing of the magnetic equatorial plane by ERG with the left side in the southern hemisphere (inside plasmapause) and the right side in the northern hemisphere (outside plasmapause).

We evaluate the wave characteristics and phase relationships associated with the PSW in the field-aligned (FA) coordinate system, in which **e**_p_ is along the background magnetic field (direction obtained from the 15-min sliding averaged data), **e**_a_ (roughly eastward) is parallel to **e**_p_ × **R** (**R** is the radial vector pointing from the centre of the Earth toward the satellite), and **e**_r_ (roughly radially outward) completes the orthogonal set. For VAP observations, the power of the perturbations of the radial magnetic field *B*_r_ peaked at the frequency of ~1.5 mHz, and ~1.4 mHz for the azimuthal electric field perturbation *E*_a_ (Fig. 2a). For ERG observations, the perturbations of the magnetic field also peaked at ~1.4 mHz (Fig. 2f). When the spacecraft arrived at the plasmapause boundary region, intense wave activities were measured. As the spacecraft left from the boundary to return to the plasmasphere, the waves quickly stopped being detected by the spacecraft (Supplementary Fig. 3). These measurements are in general agreement with the conception of a surface wave along the plasmapause boundary^24^.

Clear coherent phase relationships are found between the magnetic and electric fields in the bandpass-filtered signals (Fig. 2b–d): *B*_r_ was nearly in antiphase with *E*_a_ (−164 ± 9°) and was roughly orthogonal to the field-aligned magnetic perturbation *B*_p_ (108 ± 22°); *B*_p_ had a ~90° phase difference with both *E*_a_ (89 ± 15°) and the radial ion velocity *V*_r_ (89 ± 13°); and *B*_a_ was roughly in quadrature with *E*_r_ (83 ± 15°). However, the phase difference was not stable at a value close to 90°. By using the simultaneously measured *B*_r_ from VAP-A and VAP-B (Fig. 2e), it is determined that the azimuthal wavelength was 10° ± 0.3°, the azimuthal propagating speed was 0.010 ± 0.001° s^−1^, and the azimuthal mode number *m* was 36 ± 1 for the wave (see the Methods section, determination of propagating speed). The different wave amplitudes observed by VAP-A and VAP-B in Fig. 2e might be caused by either the variation of relative distance between the spacecraft and the plasmapause surface, or the time varying dynamic evolution of the PSW during sunward/westward propagation (Supplementary Fig. 3). The antiphase of *n*_e_ with *B*_r_ in Fig. 2b indicates that *B*_r_ was in antiphase with the geocentric distance of the plasmapause. The magnetic perturbations exhibited a 180° phase difference on either side of the magnetic equator and also on either side of the plasmapause boundary (opposite amplitude in Fig. 2h–j), meaning that the waves at the plasmapause region are fundamental mode PSW eigenmodes^24,39^. The presence of both poloidal oscillations of *B*_r_ and *E*_a_ and toroidal oscillations of *B*_a_ and *E*_r_ shows that the observed PSW manifested a mixed poloidal and toroidal modes^40^. The finite value of *m* leads to the coexistence and joint action of these two modes. At such a moderate high-*m* (~36), the poloidal perturbations are partly field-aligned guided, and the toroidal perturbations have a compressional feature^41^. The *B*_p_ component was 90° out-of-phase with *E*_a_ (Fig. 1b) and *V*_r_ was 90° lagged behind *B*_p_ (Fig. 1d), indicating the presence of fast compressional MHD wave modes. It is known that the slow mode wave can also be coupled with shear Alfvén waves, particularly in high-β plasma environment^42^. In the plasmapause region, however, the β parameter is very low (≪0.01), the observed features, therefore, suggest that the waves associated with the PSW are coupling between fast waves with shear Alfvén waves in consideration of the sunward/westward propagation of PSW.

### Conjugated sunward/westward propagating SA

Owing to the modulation of the plasmapause by the PSW, the energetic electrons and protons that intruded into the low plasmaspheric density regions (Fig. 1d, g) were possibly scattered by the electron electrostatic cyclotron harmonic (ECH) waves^20^ and precipitated into the polar upper atmosphere to generate diffuse aurora. Images from the SSUSI instrument onboard the DMSP F17 satellite at two selected times (Fig. 1b, c, see Supplementary Fig. 4 for the entire image sequence) displayed giant undulations at the equatorward boundary of the diffuse aurora in both hemispheres, i.e., SAs, which were collocated with the sawtooth-shaped plasmapause on the equatorial plane via field line mapping, as shown in Fig. 1a. Within the limitations of the temporal evolution and spatial coverage of SSUSI, it is approximately estimated that the SA was possibly initiated between 11:15 and 11:45 UTC and ended at ~15:10 UTC (Supplementary Fig. 4b, f).

**Fig. 3:**
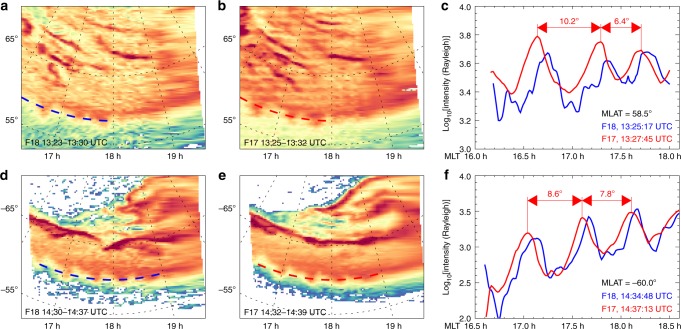
Determination of the phase speed and azimuthal wavelength of SA. **a** Auroral image obtained by the DMSP F18 satellite for 13:23–13:30 UTC in the NH with the SA captured at 13:25:17 UTC. **b** Auroral image obtained by the DMSP F17 satellite between 13:25–13:32 UTC in the NH with the SA captured at 13:27:45 UTC. **c** Emission intensity variations of the SA at the AACGM latitude of 58.5° as indicated by the blue dashed line in (**a**) and red dashed line in (**b**). **d** Auroral image obtained by the DMSP F18 satellite between 14:30 and 14:37 UTC in the SH with the SA captured at 14:34:48 UTC. **e** Auroral image obtained by the DMSP F17 satellite between 14:32 and 14:39 UTC in the SH with the SA captured at 14:37:13 UTC. **f** Emission intensity variations of the SA at the AACGM latitude of −60° as indicated by the blue dashed line in (**d**) and red dashed line in (**e**).

The DMSP F17 and F18 satellites^35^ passed through the polar region successively in ~2 min, which is perfect for analysing the dynamical evolution of the SA. With a cross-correlation analysis^43^ between the emission intensity profiles at AACGM latitudes of 58.5° in the Northern Hemisphere (Fig. 3a, b) and −60.0° in the Southern Hemisphere (Fig. 3d, e), we determined that the SA propagated sunward/westward at a phase speed of 0.01 ± 0.001° s^−1^ and the azimuthal wavelength was about 6.4°–10.2° for the SA (Fig. 3c, f). In addition, the azimuthal wavelength was shown to decrease with increasing MLT. The azimuthal wavelength and sunward/westward phase speed of the SA found from the DMSP data are in agreement with the PSW (~9.7°–10.5° and 0.01° s^−1^) obtained based on the VAP measurements, unambiguously proving that the SA was driven by the PSW; in other words, the SAs are the optical atmospheric/auroral manifestation of the PSWs.

### Poleward and sunward/westward propagating ULF waves

**Fig. 4:**
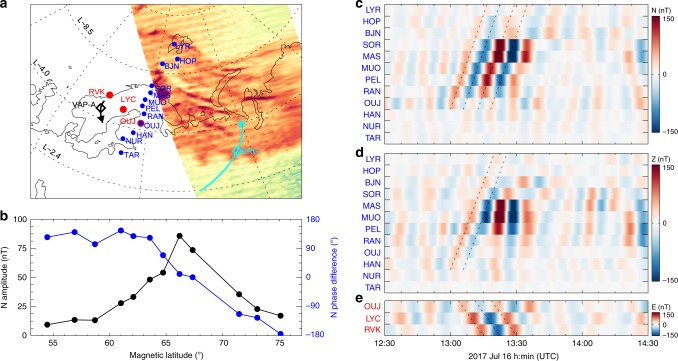
ULF waves and FLR observed on the ground. **a** Stations selected from the IMAGE magnetometer array with the background auroral image observed by DMSP F17 satellite at 13:27:45 UTC (see also Fig. 1b). The blue chain is aligned in almost the same magnetic longitudes of ~106 ± 4° with the magnetic latitude increasing from 54.5° at TAR to 75.1° at LYR. The red chain is aligned in almost the same magnetic latitudes of ~61.5 ± 0.5° with the magnetic longitude increasing from 93.3° at RVK to 106.1° at OUJ. The black/cyan arrow represents the footpoints of VAP-A/ERG satellite moving towards low/high latitudes between 13:40 UT and 15:00 UT with the diamond marking plasmapause crossing. **b** Amplitude (black) and phase difference (blue) of the N component of the blue chain plotted as a function of geomagnetic latitude. **c** Bandpass filtered (1.4 ± 0.5 mHz) N component (north) of the geomagnetic field perturbations. **d** Bandpass filtered (1.4 ± 0.5 mHz) Z component (vertical) of the geomagnetic field perturbations. Clear poleward propagation of the waves is highlighted by the dashed lines. **e** Bandpass filtered (1.4 ± 0.5 mHz) E component (east) of the geomagnetic field perturbations. Clear sunward/westward propagation of the waves is highlighted by the dashed lines.

Apart from driving the SA, the PSW generated outward-propagating ULF waves outside the plasmapause, giving rise to the field line resonance (FLR)^3^ and driving ULF geomagnetic pulsations. Figure 4 shows the analysis of magnetic perturbations observed by ground-based station chains (Fig. 4a) from the International Monitor for Auroral Geomagnetic Effects (IMAGE) magnetometer array^44^. The power spectra of the magnetic field perturbations showed several significant signals at ~0.6 mHz, ~1.1 mHz, ~1.4 mHz, and ~2.0 mHz, with lower frequency pulsations occurring at higher latitudes (Supplementary Fig. 5). The wave amplitude of the N (north) component in the frequency of 1.4 ± 0.5 mHz (the closest to the frequency of PSW-associated waves in Fig. 2a) maximized at a magnetic latitude of ~66° (MAS station), while the phase changed by nearly 180° over the amplitude maximum (Fig. 4b), indicating the driving of FLR outside the plasmapause by the PSW. The 1.4 ± 0.5 mHz bandpass filtered N (Fig. 4c) and Z (Fig. 4d) components of the geomagnetic perturbations showed clear poleward propagation (i.e., radially outward in the magnetosphere) of the ULF waves starting from the OUJ station (at the AACGM latitude of 61.42° and longitude of 105.46°), which are the closest to the SA (Fig. 4c, d), i.e., the counterpart to the sawtooth-shaped plasmapause region in the magnetosphere. This is apparently contrary to the inward (or equatorward) propagation of the ULF waves generated by external sources (e.g., magnetopause^15^ or in the near-Earth magnetotail^14,45^). The 1.4 ± 0.5 mHz bandpass filtered E component of the geomagnetic perturbations (Fig. 4e) showed clear sunward/westward propagation of the ULF waves with a speed of ~0.012° s^−1^ and an azimuthal mode number of 36.8 ± 0.6 (see the Methods section, determination of *m* on ground). Both the propagating speed and the azimuthal mode number are consistent with those of the PSW and the SA.

## Discussion

The 16 July 2017 geomagnetic storm provided an opportunity to identify the PSW and its consequences of oscillating both the magnetosphere and the diffuse aurora. The enhanced convection electric field during the storm made the plasmapause in the afternoon-dusk sector very sharp. Inhomogeneities in the plasma density and magnetic field lead to the coupling between the shear Alfvén waves and the magnetosonic waves on closed field lines. Field oscillations in the magnetosphere usually have a continuous spectrum. Nevertheless, solution of the MHD wave equation shows the coupling between the surface wave (i.e., evanescent compressional Alfvén wave) and the shear Alfvén wave if there exists a sharp change in Alfvén speed (related to changes in magnetic field and plasma mass density), and a localized perturbation propagating along the plasma surface across which the Alfvén speed changes will suddenly possess a discrete eigenfrequency^24^. Since the resonant absorption is negligibly small^46^, a localized coupled ULF wave corresponding to the discrete eigenmode can thus be excited at such a location by an impulse which has a frequency spectrum that covers this eigenfrequency.

Observations in both the topside ionosphere and the plasmasphere showed that the radial width of the plasmapause was ~0.1–0.2 *R*_E_ (see Supplementary Figs. 6, 7), which was much smaller than the transverse wave scale of ~0.8 *R*_E_ for the PSW with *m*-value 36 at a geocentric distance of 4.5 *R*_E_, satisfying the requirement of the interface width in exciting surface waves^47^. The sharp discontinuity in the density led the magnetosonic wave to be undamped^24,46,48^, allowing excitation of a standing surface eigenmode, i.e., the PSW propagating sunward/westward at the plasmapause boundary and along the field lines to both the northern and southern polar ionosphere. Using the plasmapause crossing measurements, it is estimated that the frequency of the fundamental poloidal wave is 1.35–2.11 mHz, close to 1.5 mHz (see the Methods section, estimation of eigenfrequency). Above estimations indicate that the configuration of the plasmapause during the storm main phase could provide favorable conditions to excite PSW.

The PSW could be excited by external perturbations such as the sudden enhancement of the plasma pressure in the duskside ring current/plasma sheet owing to fast storm time injections. The driving sources could be either monochromatic with a frequency similar to the eigenfrequency of the plasmapause surface or impulsive and broadband whose frequency range covers the eigenfrequency of the plasmapause surface. Between 11:00 and 11:30 UT, strong ion/electron injections were observed (Supplementary Figs. 8, 9). The power spectrum density of the energetic ion/electron flux between 11:00 and 13:00 UT exhibits a clear peak at 1.5 mHz (Supplementary Fig. 10), close to the eigenfrequency of the plasmapause surface. Such a periodically enhancing particle flux led to impinging of periodically varying plasma pressure on the plasmapause surface and may actually excite the PSW at 1.5 mHz. In addition, it is noted that other types of pressure variations, such as impulsive injection (like a delta function) and continuous injection (like a step function), are both broadband in frequency domain. Take the magnetopause surface wave for an example, the magnetosheath jet’s total pressure is impulsive and broadband^9^. Therefore, impulsive or continuous injection may both contribute to the excitation of the PSW. The proposed excitation process of PSW is similar to the magnetopause surface waves excited by the sudden enhancement of solar wind pressure^6^, interplanetary shocks^49^, or magnetosheath jets^9^. The free energy should be continuously supplied to maintain the long-term evolution and propagation of the PSW. Consistency between the durations of the PSW (or SAs) and the enhanced hot plasma pressure (Supplementary Fig. 9f) indicates that the free energy was provided by the hot plasma injections. Other internal instabilities, such as the ion drift resonances which can excite fundamental poloidal waves^50,51^ and the Kelvin-Helmholtz instability which can excite magnetopause surface waves^7,8^, could certainly play a role at some point in the excitation of the ULF waves, in addition to the PSW-associated ULF waves. Drift wave perturbations or the effect from ULF waves in the vicinity of the plasmapause might also be possible interpretations to the observed waves. Multi-satellite measurements show that the observed waves propagated along the plasmapause surface with a fixed frequency, excited outward-propagating ULF waves and drove FLR outside the plasmapause. These characteristics are similar to that of the magnetopause surface waves^8,9^ which excite earthward-propagating ULF waves and drive FLR in the magnetosphere. In these regards, we suggest that the PSW concept should be a reasonable interpretation for the observations.

The equatorial plasmapause became sawtooth-shaped due to the modulation of the PSW. The low-density region of PSW was intruded by hot plasma. Scattering and precipitation of the hot plasma by waves like ECH then generated SA in the afternoon-evening sector, which had the same azimuthal wavelength and sunward/westward phase speed as the PSW. In addition, the local lower ratio of electron plasma frequency to gyrofrequency in the night sector during disturbed periods also leads to much more efficient scattering by chorus waves (effective for ECH as well) just outside the plasmapause^52–54^. The SAs that occur at the equatorial boundary of the diffuse aurora on the dusk side are different from other quasi-periodic auroral structures such as the torch auroral structures (or omega band)^55^ and the pulsating auroral forms generated by giant pulsations^56^, both of which occur in the auroral oval and in the midnight to morning sector, although similar wave activities are found to exist during these different auroral morphologies.

Like the magnetopause surface wave that generates earthward-propagating ULF waves and drives FLR in the magnetosphere^8^, the PSW generated outward-propagating ULF waves and drove FLR outside the plasmapause. Such a process has not yet been observed and interpreted in the existing literature. The amplitude and phase structures on the ground are different from the giant pulsations though they have similar wave number and structures^57^. The amplitude and phase structures observed here are due to the radially outward propagation of the ULF waves from the plasmapause boundary and resonance with local field lines on the duskside, while the giant pulsations are latitudinally localized and occur almost exclusively on the morningside with peak occurrence in the postmidnight sector^58^. The ground signatures of surface waves are not well understood, including both the PSW and the magnetopause surface wave^9^. In this study, we show observational evidence that PSW can exhibit different signals to the conjugated ground magnetometer data. Further theoretical investigations, simulations, and observations are necessary to characterize the occurrence rate and properties of PSW.

**Fig. 5:**
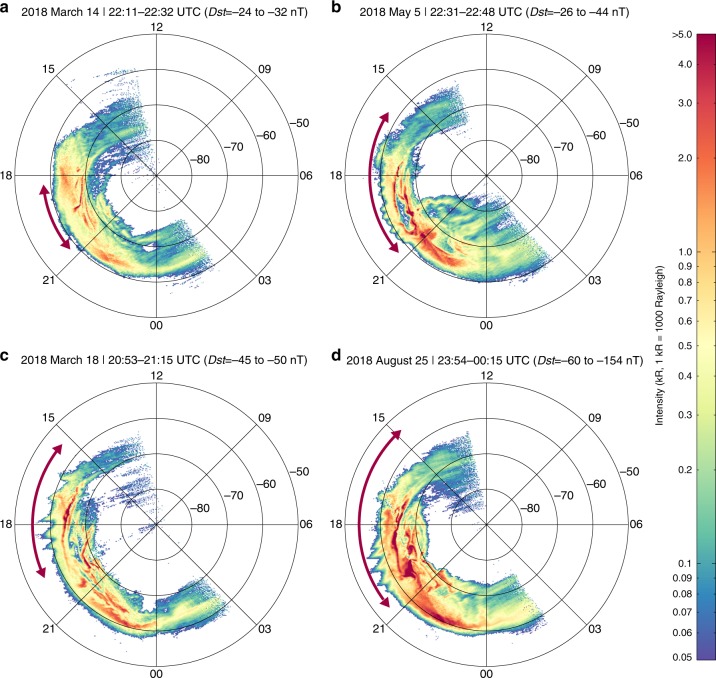
SAs observed by FY-3D WAI during four geomagnetic storms in 2018. All the images observed between **a** 22:11–22:32 UTC on 14 March 2018, **b** 22:31–22:48 UTC on 5 May 2018, **c** 20:53–21:15 UTC on 18 March 2018, and **d** 23:54–00:15 UTC on 25 August 2018 are projected onto a reference sphere at a height of 110 km in the AACGM–MLT coordinate system. The SAs are highlighted by the red arrows. Detailed structural parameters of the SAs are listed in Table 1.

Although the PSWs are hardly captured by in situ observations since satellites are required to cross the plasmapause region at a specific time and at a certain location, the SAs that visualize the PSW are frequently observed during geomagnetic storms. Besides the DMSP satellites, the recently launched Chinese Fengyun-3D (FY-3D) satellite in a low-Earth orbit can capture high-resolution global images of SAs (Fig. 5) with the on-board wide-field auroral imager (WAI)^59^. We have surveyed the aurora data recorded by both DMSP and FY-3D satellites during geomagnetic storms (the minimum disturbance storm-time (Dst) index less than -40 nT) from 2014 to 2018 (listed in Supplementary Table 1). The probability of occurrence of SAs is found to be greater than 90% during geomagnetic storms (94 out of 103, as shown in Supplementary Table 1). For the remaining 9 storms, it is not clear whether these auroral structures are present or not due to the spatial coverage and temporal evolution of the auroral images. Furthermore, coordinated ground-based geomagnetic data were available in the dusk sector (MLT = 16–19 h) for 24 events of the 94 observed SAs. The geomagnetic pulsations occurred simultaneously with the SAs and propagated essentially radially outward when mapping to the magnetic equator and the amplitude peaked outside the plasmapause (Supplementary Fig. 11). These results definitely indicate that the PSWs, as well as the SAs, are systematic and crucial consequences of geomagnetic storms. The reconfiguration and energy redistribution processes in the magnetosphere and ionosphere associated with the PSW appear to be common and regular during the geomagnetically disturbing times, and theoretical and statistical studies are needed in the future for better understanding of the rules that govern these processes.

The high resolution auroral images from the FY-3D satellite show that the AACGM latitudes of crest, azimuthal wavelengths, and crest-to-trough amplitudes of the SAs vary with the strengths (i.e., the Dst values) of storms (Table 1), implying that the characteristics of the PSWs also vary with geomagnetic activity and that the following magnetospheric and ionospheric effects will also be different. Although it remains unclear what mechanisms determine the azimuthal wavelength (i.e., azimuthal mode number) of the PSW from a theoretical view, these observations could provide important implications to future theoretical investigations on this topic. Understanding the factors (e.g., configuration of the plasmapause boundary and the plasma pressure variations outside the plasmapause during storms) that trigger and control the generation and evolution of the PSW is critical in establishing the generation process. In addition, the wave-particle interactions that lie behind this process are key to understanding the resultant energy transfer and auroral activities.

**Table 1:** Structural parameters of the SAs extracted from Fig. 5.

Panel	Dst index (nT)	AACGM latitude of crest (°)	Azimuthal wavelength (°)	Crest-to-trough amplitude (°)
Fig. 5a	−24 to −32	−61.4	4.6	1.8
Fig. 5b	−26 to −44	−59.3	3.4 to 5.3	1.8 to 4.4
Fig. 5c	−45 to −50	−57.7	5.4 to 12.8	4.9 to 7.1
Fig. 5d	−60 to −154	−55.0	5.1 to 8.1	3.8 to 8.8

The generation of surface waves and ULF waves are fundamental plasma processes in space environments and can occur in other planetary magnetospheres^3,60,61^. As is well-known in the space of giant planets, the corotation breakdown (the cause of the plasmapause in the Earth) is known to be fundamental to the dynamics of the rapid rotating planetary magnetospheres (e.g., Jupiter and Saturn)^62,63^ with the brightest auroral emissions associated with this process^64^. Therefore, the processes identified along terrestrial plasmapause boundary layer may be even more critical to the environments at rapidly rotating planets and may provide a crucial direction for investigations.

## Methods

### Coordinate systems

The geocentric solar magnetospheric (GSM) coordinate system is defined as follows^65^. The *x*-axis of GSM points from the Earth to the Sun, the *z*-axis is the projection of dipole axis on geocentric solar ecliptic (GSE) yz plane (the *x*-axis of GSE points from the Earth to the Sun, the *z*-axis of GSE points to the ecliptic north pole, the *y*-axis of GSE completes the right-handed system), and the *y*-axis of GSM completes the right-handed system. The *y*-axis of the solar magnetic (SM) system is perpendicular to the plane containing the Sun-Earth line and the dipole axis, the *z*-axis is along the dipole axis, and the *x*-axis completes the right-handed system. The modified GSE (mGSE) coordinate system is a near GSE system for Van Allen Probes (VAP). The *x*-axis of mGSE is the spin axis unit vector in GSE coordinates, the *y*-axis is anti-parallel to the cross product between the *x*-axis of mGSE and the *y*-axis of GSE, and the *z*-axis of mGSE completes the right-handed system. Altitude-adjusted corrected geomagnetic (AACGM)^38^ coordinates are an extension of corrected geomagnetic coordinates that more accurately represent the actual magnetic field. In AACGM coordinates points along a given magnetic field line are given the same coordinates and are thus a better reflection of magnetic conjugacy.

### Data usage

Observations reported in this paper are taken from the VAP-A and VAP-B spacecraft, the DMSP F17 and F18 satellites, the ERG spacecraft, the FY-3D satellite, the Time History of Events and Macroscale Interactions during Substorms (THEMIS) E spacecraft, the Magnetospheric Multiscale (MMS) mission, the Geostationary Operational Environmental Satellite (GOES) 15 satellite, the ground-based IMAGE magnetometer array, and the SuperMAG database. Detailed information is introduced below.

The spectrogram (6 s resolution, 10 kHz to 500 kHz) from the high frequency receiver of the Electric and Magnetic Field Instrument Suite and Integrated Science (EMFISIS)^30^ onboard VAP and the spectrogram (1 s resolution, 10 kHz to 10 MHz) from the high frequency analyzer of the Plasma Wave Experiment (PWE)^32^ onboard ERG are used to determine the plasmaspheric electron density.

The magnetic field (1 s resolution in GSM coordinates) from the fluxgate magnetometer of EMFISIS^30^ and the electric field (32 sample/s in mGSE coordinates) from the Electric Field and Waves (EFW) instrument^66^ onboard VAP and the magnetic field (8 s resolution in GSM coordinates) from the Magnetic Field Experiments (MGF)^67^ onboard ERG are used together to evaluate the wave characteristics associated with the plasmapause surface wave. For VAP spacecraft, the spin axis electric field component (*E*_x_) is estimated using the assumption that that **E** • **B** = 0 or the parallel electric field is zero. This is used most frequently for large-scale convection electric fields, MHD structures and ULF waves, and small-scale waves for which perpendicular electric fields are larger than parallel. Then the ion velocity is calculated by **V** = **E** × **B** /*B*^2^.

The omni-direction energy flux data from the Helium, Oxygen, Proton, and Electron (HOPE) mass spectrometer^31^ onboard VAP, from the low-energy particle experiments–electron analyzer (LEP-e)^33^ and the low-energy particle experiments–ion analyzer (LEP-i)^34^ onboard ERG, from the Fly’s Eye Energetic Particle Sensor (FEEPS)^68^ and the Hot Plasma Composition Analyzer (HCPA)^69^ onboard MMS-1, and from the Energetic Particle Sensor (EPS)^70^ onboard GOES-15 are used to evaluate the distributions and evolutions of hot plasma.

The auroral disk images in N_2_ Lyman–Birge–Hopfield (LBH) bands from the Special Sensor Ultraviolet Spectrographic Imager (SSUSI)^35^ onboard DMSP F17 and F18 satellites are used to acquire the parameters of the SA. All the auroral images are projected onto a reference sphere at a height of 110 km. Both the DMSP F17 and F18 satellites orbit the Earth in a sun-synchronous orbit with a period of ~102 min and two disk images of aurora are obtained in each orbit. All the auroral images during the PSW event are shown in Supplementary Fig. 4. On 16 July 2017, the DMSP F17 and F18 satellites passed the same polar region successively with a time difference of ~2 min, and therefore, only the F17 images are shown in Supplementary Fig. 4. The densities of O^+^, H^+^, and He^+^ measured by the Retarding Potential Analyzer (RPA)^71^ on board the DMSP satellite are used to calculate the latitudinal plasma mass density profiles, which can reflect the radial profile of the plasma mass density in the plasmasphere. Examples are shown in Supplementary Fig. 7.

The wide-field auroral imager (WAI)^59^ onboard FY-3D satellite, which was launched on 15 November 2017 into a sun-synchronous orbit at an altitude of ~840 km with a period of ~102 min, also provided auroral disk images in N_2_ LBH bands but with larger field-of-view that can capture the global high-resolution structure of the SA (Fig. 5).

The geomagnetic field data (10 s resolution) from the ground-based station chains (latitudinal chain from low to high latitude: TAR, NUR, HAN, OUJ, RAN, PEL, MUO, MAS, SOR, BJN, HOP, and LYR; longitudinal chain from west to east: RVK, LYC, and OUJ) of the IMAGE magnetometer array^44^ and the geomagnetic field data (1 min resolution) from the ground-based station chain (from low to high latitude: OTT, T51, T30, T52, T45, T31, T44, T46, and T47) of the SuperMAG database^72^ are used to evaluate the geomagnetic pulsations on the ground. The latitudinal IMAGE chain is aligned in almost the same magnetic longitudes of ~106 ± 4° (in the dusk sector at ~15:00 UTC) with the magnetic latitude increasing from 54.5° at TAR to 75.1° at LYR. The longitudinal IMAGE chain is aligned in almost the same magnetic latitudes of ~61.5 ± 0.5° with the magnetic longitude increasing from 93.3° at RVK to 106.1° at OUJ, and the SuperMAG chain is aligned around magnetic longitudes of 0 ± 4° (in the dusk sector at ~23:00 UT) with the magnetic latitude increasing from 55.0° at OTT to 71.5° at T47. All the geomagnetic field data are presented in the NEZ frame, in which horizontal components N and E point geomagnetically north and east, respectively, and Z is the vertical component. The original data are all detrended by subtracting the 1-h sliding averages.

### Calculation of plasmaspheric electron density

The electron density (*n*_e_, in cm^–3^) measured by VAP spacecraft^73^ is calculated by *n*_e_ = (*f*^2^_UHR_ – *f*^2^_ce_)/8980^2^, where *f*_UHR_ is the upper hybrid resonance (UHR) frequency in Hz identified from the frequency-time spectrogram of electric field of the EMFISIS instrument, *f*_ce_ = *eB*/*m*_e_ is the electron cyclotron frequency in Hz, *B* is the strength of the magnetic field simultaneously measured by the EMFISIS instrument, and *m*_e_ is the electron mass. The method for the ERG spacecraft is the same. The inferred electron densities are shown in Fig. 1e, f and h, i. For the THEMIS-E satellite, the spacecraft potential (refers to the potential of the spacecraft body relative to the ambient plasma) measured by the electric field instrument (EFI)^74^ and the electron thermal velocities measured by the electrostatic analyzer (ESA)^75^ are used to calculate the electron density, which is used to evaluate the plasmapause configuration (Supplementary Fig. 6).

### Determination of the azimuthal propagating speed of PSW

We define the azimuthal wavelength of the PSW as *λ*_PSW_ and the azimuthal propagating speed as *v*_PSW_. Between 13:50 and 14:40 UTC, the longitudinal separation between VAP-A and VAP-B is *dλ*_sat_ = 17.3 ± 0.3° and the azimuthal angle difference between the electro density peaks observed by VAP-A is *dλ*_peak_ = 3.0 ± 0.2° with a time interval of *dt* = 725 ± 25 s (Fig. 2b). Since VAP-A is closer to dusk than VAP-B, the phase difference indicates a sunward/westward propagating wave, and *λ*_PSW_ should be less than *dλ*_sat_ and greater than *dλ*_peak_. The phase of *B*_r,VAP-A_ leading *B*_r,VAP-B_ by 90 ± 1° between 13:45 and 14:15 UTC (Fig. 2e) indicated that *dλ*_sat_ = (3/4 + *n*) *λ*_PSW_ (*n* = 0, 1, 2, …). The loss of phase after 14:15 in Fig. 2f is because the VAP-B spacecraft had entered the plasmasphere and was far away from the plasmapause region. For *n* = 0, we get an azimuthal mode number *m* ≈ 16 and *λ*_PSW_ = 22.5°, which dissatisfies the limitation on *λ*_PSW_. When *n* = 1, we get *m* = 36 ± 1 and *λ*_PSW_ = 10 ± 0.3°, which satisfies the limitation on *λ*_PSW_. According to the relationship *λ*_PSW_ = *dλ*_peak_ + *v*_PSW_ × *dt*, we obtain the sunward/westward propagating speed *v*_PSW_ = 0.01 ± 0.001° s^−1^. Both the azimuthal wavelength and the sunward/westward propagating speed showed agreement between the PSW (~9.7°–10.5° and 0.01° s^−1^) and the SA (~6.4°–10.4° and 0.01° s^−1^), unambiguously proving that the SAs are driven by the PSWs, i.e., the SA is the optical manifestation of the PSW. Because the PSW was observed at an earlier MLT than the SA, and the wavelength of the PSW is generally greater than that of the SA, which is consistent with the decrease in SA wavelength with increasing MLT (Fig. 3).

### Determination of the azimuthal wave number on the ground

The three stations (OUJ, LYC, and RVK) are used to calculate the *m*-value of the sunward/westward propagating ULF waves on the ground. The azimuthal mode number *m* of the ULF waves is calculated by *m* = *dφ*/*dϕ*, where *dφ* is the phase angle difference of the ULF waves between two stations and *dϕ* is the magnetic longitude separation between two stations. Using cross-phase analysis between station pairs (OUJ-LYC and OUJ-RVK, *dϕ*_OUJ-LYC_ = 6.85°, *dϕ*_OUJ-RVK_ = 12.83°, *dφ*_OUJ-LYC_ = 248°, and *dφ*_OUJ-RVK_ = 480°) during 13:00 UT and 13:30 UT, the *m*-value is calculated to be 36.8 ± 0.6, consistent with the *m*-value of the PSW. The averaged time difference of the wave peaks and averaged magnetic longitude difference between the station pairs are *dt* = ~400 s and *dϕ* = 6.42°, respectively. Considering the Earth’s eastward rotating speed of 0.0042° s^−1^, the sunward/westward propagating speed of the ULF waves is ~0.012° s^−1^. It is noted that the direction of ULF wave propagation can be affected in the data by the shape of the plasmapause, and the propagation direction and speed can be more precisely determined if more station chains at different latitudes are available at the same time.

### Estimation of the eigenfrequency at the plasmapause

According to the dispersion equation given by Chen and Hasegawa^24^, the wave propagation speed along the magnetic field line at the plasmapause is *v* = 2^1/2^*v*_A_, where *v*_A_ is the Alfvén speed of the magnetic field line inside the plasmapause boundary. According to THEMIS-E satellite measurement (Supplementary Fig. 6), the strength of the magnetic field is *B* = ~400 nT and the electron number density (equivalent to total number density of H^+^, He^+^, and O^+^) is ~1500 cm^−3^ inside the plasmapause boundary. Since no ion composition measurements were available in the magnetosphere during this storm, typical values of relative ion concentrations in literature are considered, with the relative concentration of O^+^ between 5 and 30% and He^+^ between 5 and 10%^17^. The plasma mass density is estimated to be 4.54 × 10^−18^~1.11 × 10^−17^ kg m^−3^ and the resultant *v* is 150 ~ 240 km s^−1^. The length of the field line *Λ* at the plasmapause is calculated to be ~5.6 × 10^4^ km with the Tsyganenko 96 magnetic field model. Taking into account that half a wavelength equals to *Λ* for the fundamental poloidal wave, we get the fundamental frequency ω = *v*/(2*Λ*) = 1.35 ~ 2.11 mHz, close to 1.5 mHz. The above estimations indicate that the configuration of the plasmapause during the storm main phase supports the excitation and generation of the PSW.

## Supplementary information

Supplementary Information

## Data Availability

DMSP SSUSI data in format of SDR-DISK are available at https://ssusi.jhuapl.edu/. All the WAI raw data are processed and provided by the ground application system at National Satellite Meteorological Center, China Meteorological Administration. The EMFISIS instrument data are obtained from the University of Iowa at the website http://emfisis.physics.uiowa.edu/data/index. All HOPE data are available at the website http://www.RBSP-ect.lanl.gov/. The EFW data are available at http://www.space.umn.edu/rbspefw-data/. Science data of the ERG (Arase) satellite were available from the ERG Science Center operated by ISAS/JAXA and ISEE/Nagoya University (https://ergsc.isee.nagoya-u.ac.jp/index.shtml.en). The MMS satellite data are available at the MMS Science Data Center at https://lasp.colorado.edu/mms/sdc/public/. The THEMIS mission data are available at http://themis.ssl.berkeley.edu/data_retrieval.shtml. The DMSP RPA data and the GOES EPS data are available at the NOAA National Centers for Environmental Information at https://satdat.ngdc.noaa.gov/. The IMAGE magnetometer data are available at http://space.fmi.fi/image/www/index.php?page=home. The SuperMAG data are available at http://supermag.jhuapl.edu/mag/). The solar wind parameters are available from NASA OMNIWeb (https://omniweb.gsfc.nasa.gov/). The geomagnetic indices are available from World Data Center for geomagnetism, Kyoto at http://wdc.kugi.kyoto-u.ac.jp.

## Code availability

The SPEDAS software used for wave analysis is available at http://themis.ssl.berkeley.edu.
