# Supplementary material for: Plasmapause surface wave oscillates the magnetosphere and diffuse aurora
Source: Nat Commun. 2020 Apr 3;11:1668. doi: 10.1038/s41467-020-15506-3 (PMC7125146; doi:10.1038/s41467-020-15506-3)
Supplement: Supplementary file 1 — Supplementary Information [file 41467_2020_15506_MOESM1_ESM.pdf]

Supplementary Information for

Plasmapause Surface Wave Oscillates the Magnetosphere and Diffuse Aurora

He et al.

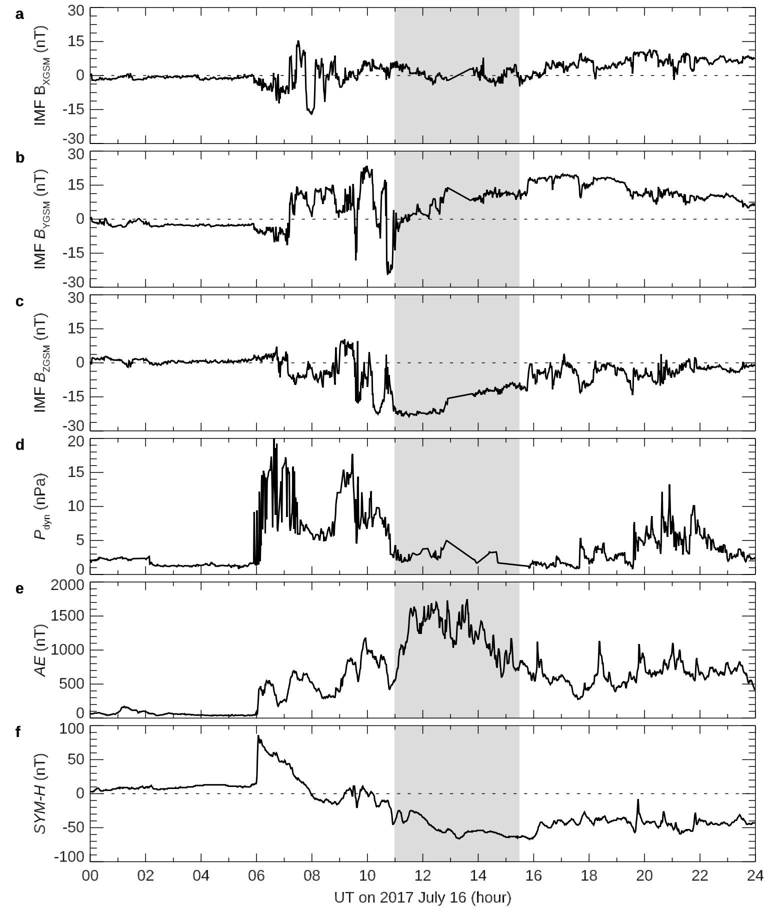

Supplementary Figure 1. Solar wind and geomagnetic conditions on 16 July 2017. a-c The *x*, *y*, and *z* components of the interplanetary magnetic field (IMF) in GSM coordinate system. **d** The solar wind dynamic pressure. **e** *AE* index. **f** *SYM-H* index. The grey region indicates the period when SA and PSW were observed.

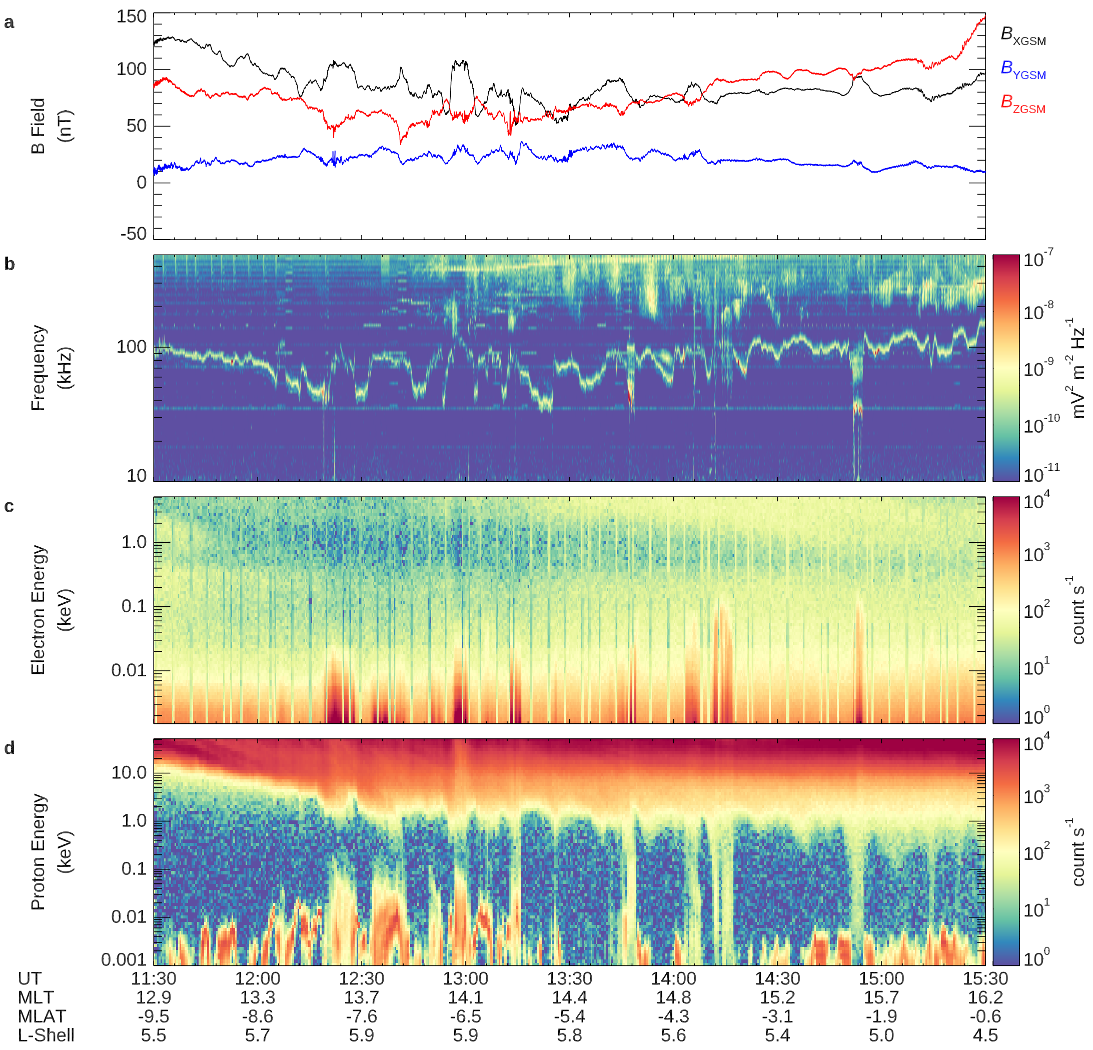

Supplementary Figure 2. Observations by VAP-B spacecraft on 16 July 2017. a The *x* (black), *y* (blue), and *z* (red) components of the magnetic field in GSM coordinate system. **b** Spectrogram of the electric field from the high frequency receiver of the EMFISIS. The next two panels show the energy spectrograms of electrons **c** and protons **d** measured by the HOPE mass spectrometer, respectively.

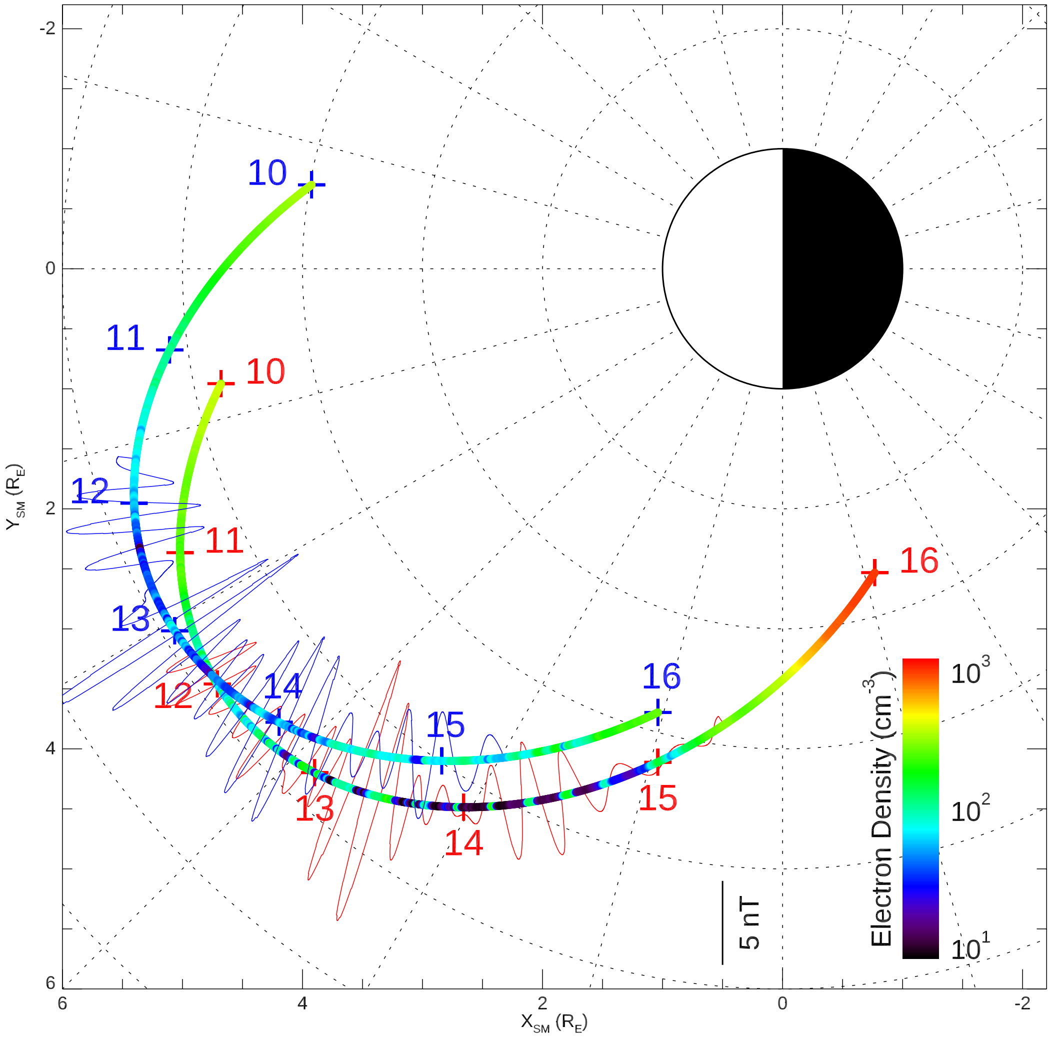

Supplementary Figure 3. VAP-A and VAP-B satellites observations on July 16 2017. The crosses denote the locations of the VAP-A (red) and VAP-B (blue) satellites in one-hour interval from 10:00 UT to 16:00 UT. The logarithmically color-coded electron density distributions are overlaid on the trajectories with the color bar shown at the lower right corner. Also overlaid on the trajectories are the radial component of the magnetic field perturbations (1-2 mHz filtered) measured by the VAP-A (red) and VAP-B (blue) satellites with the scale bar shown at the lower right corner.

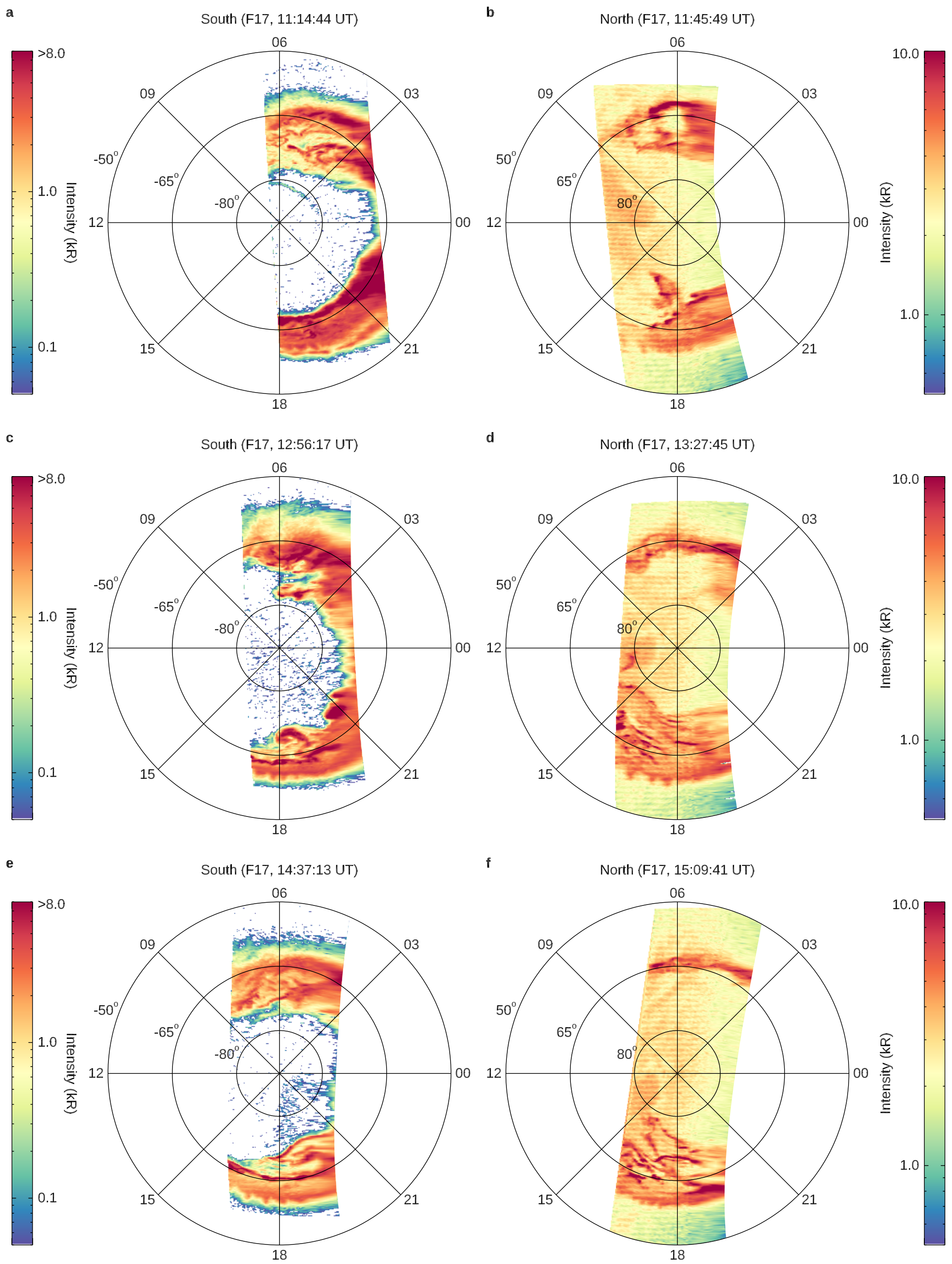

Supplementary Figure 4. Auroral images observed by DMSP F17 satellite. Since the F17 and F18 satellites passed the auroral region almost simultaneously, only F17 images are shown in panels **a-f**. The times when the satellite pass the equatorward boundary of the duskside auroral oval are shown at the top of each panel. All the images are the electron auroral images in N2 LBH bands. Actually, same SAs also appeared in auroral images at H 121.6 nm, O 135.6 nm, and O 130.4 nm wavelengths (not shown here), indicating precipitations of both energetic protons and electrons.

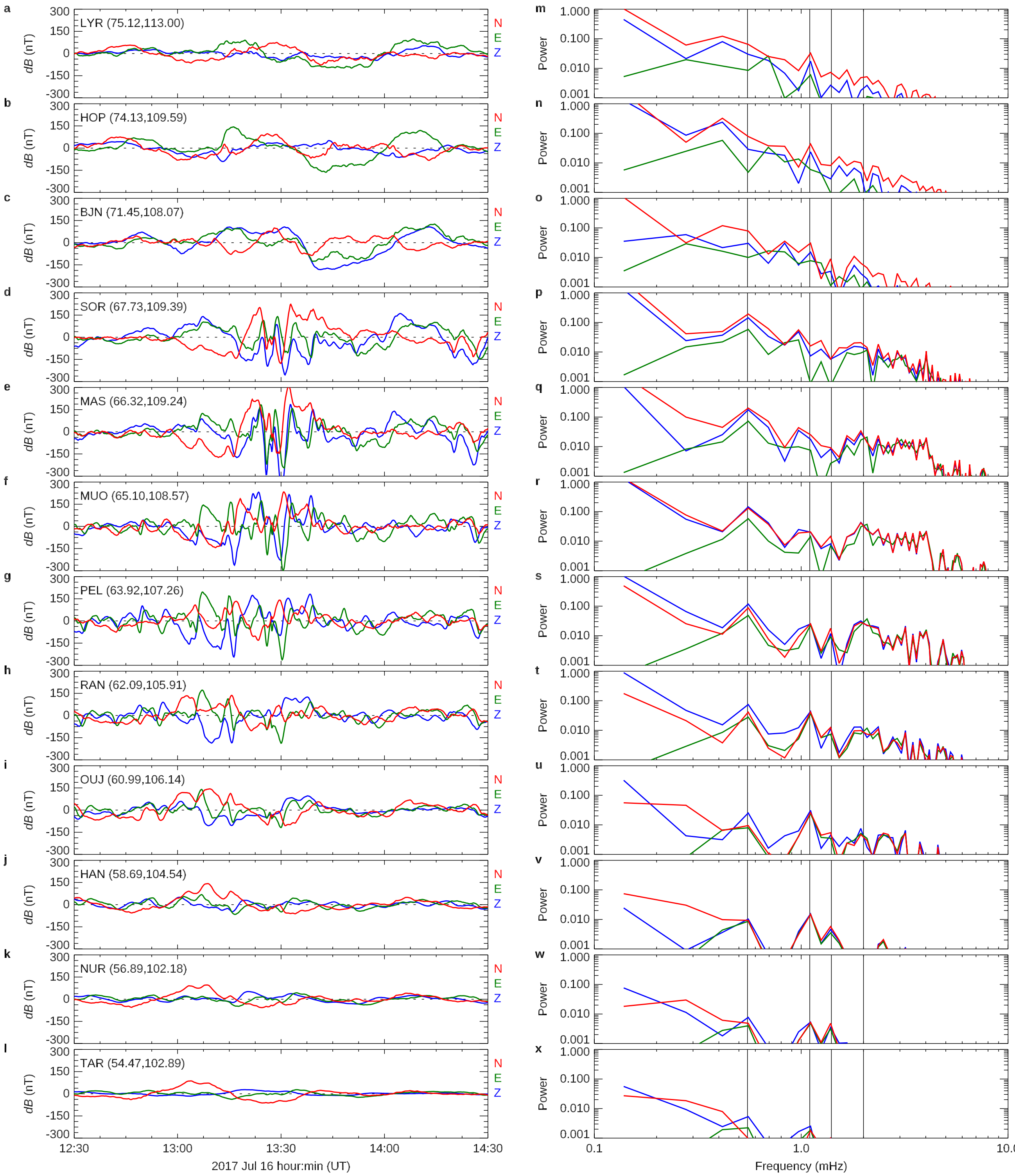

Supplementary Figure 5. Spectra analysis of the ground magnetic field perturbations. Data from the stations are presented in panels **a-l**. All the stations are aligned in almost the same magnetic longitudes of ~106°±4°. Each station is ~1°-3° apart in magnetic latitude as shown in the upper left corners of panels **a-l** (numbers in the brackets denote the magnetic latitude and longitude of the station). The locations of the stations can also be found in Fig. 4a. Data are available at 10 s resolution and presented in geomagnetic coordinates where the horizontal components N (red) and E (green) point geomagnetically

north and east, respectively, and Z (blue) is the vertical component. Panels **a-l** show the perturbations (*dB*) of the magnetic field detrended by the 1-hour sliding averages. Panels **m-x** show the normalized Fast Fourier Transform power spectra density of the magnetic field perturbations corresponding to each station shown in panels **a-l**, respectively. Significant signals at ~0.6 mHz, ~1.1 mHz, ~1.4 mHz, and ~2.0 mHz are marked by the vertical lines in panels **m-x**.

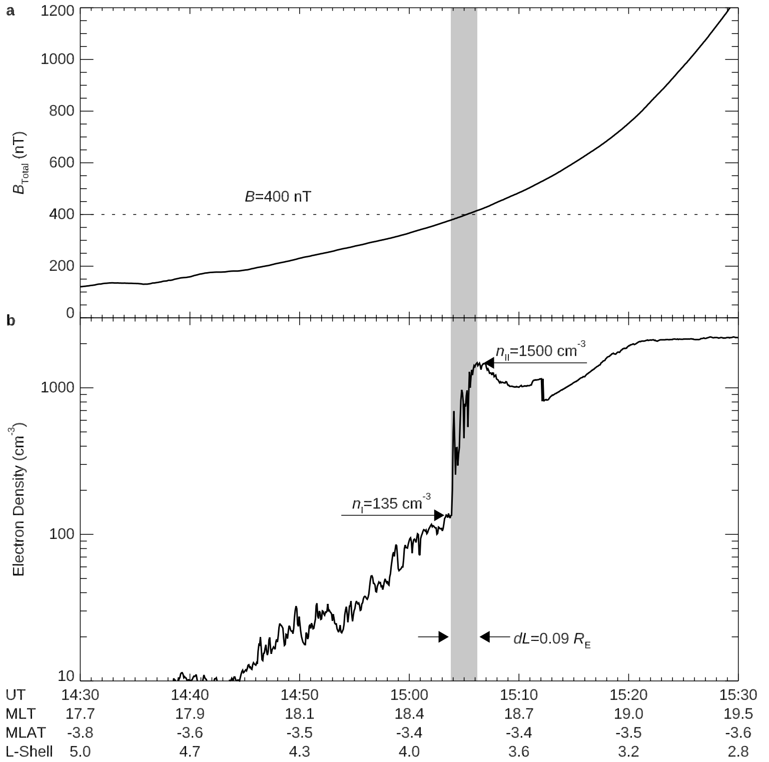

Supplementary Figure 6. THEMIS-E spacecraft observations on July 16 2017. a Magnetic field strength. **b** Electron density in the plasmasphere. The dotted line in **a** denotes the magnetic field strength of 400 nT. The grey regions indicate the plasmapause boundary.

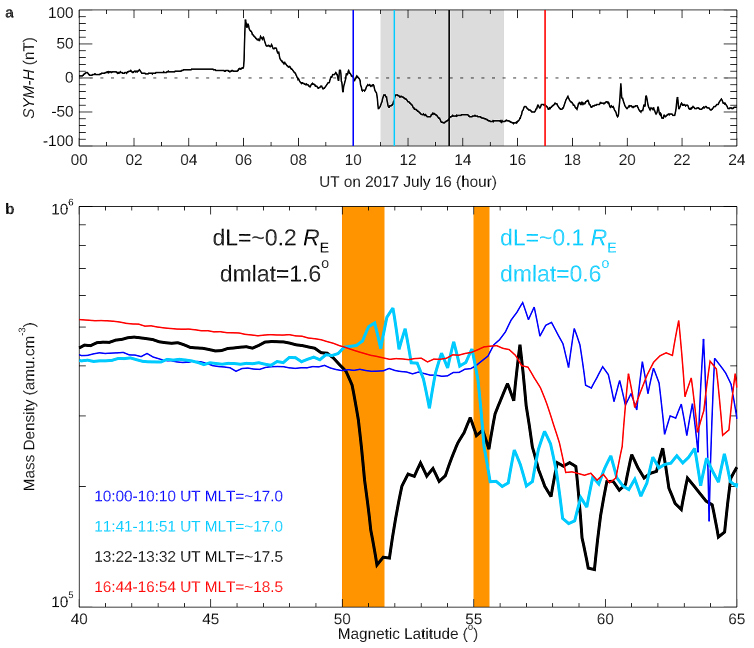

Supplementary Figure 7. DMSP F17 observations in the topside ionosphere on July 16 2017. a Temporal variation of the *SYM-H* index. The four vertical lines indicate the polar crossing for the DMSP satellite. The colors correspond to the colored curves in the bottom panel. **b** Plasma mass density versus magnetic latitude calculated from the ion composition measurements by the RPA instrument onboard DMSP F17 satellite. The two clear plasmapause structures are marked as grey color. It is shown that at the beginning of the main phase (~10:00 UT), no obvious boundary structure was presented in the density profile (blue curve). Between 11:00 UT and 12:00 UT during the main phase, when the PSW was initiated, the mass density profile showed a very sharp boundary structure (cyan curve, orange region in panel b), corresponding to a latitudinal width of 0.6° and a radial width of ~0.1 R_E when mapped onto the magnetic equatorial plane using Tsyganenko-96 magnetic field model. This indicates that the plasmapause boundary became very sharp. As the main phase continued, the density profile kept very sharp (black curve, orange region, corresponding to a latitudinal width of 1.6° and a radial width of ~0.2 R_E) and moved towards the lower latitude. In the recovery phase, the boundary returned to relatively smooth and moved towards high latitude (red), implying the recovering of the plasmasphere and smoothing of the plasmapause boundary.

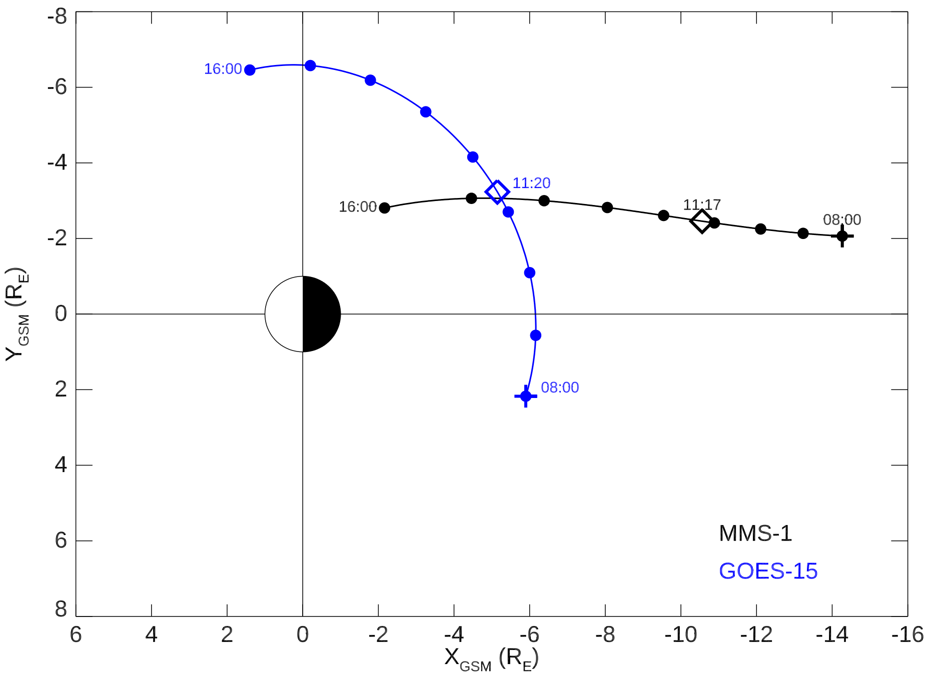

Supplementary Figure 8. Trajectories of MMS-1 and GOES-15 on July 16 2017. The dots in the curves are drawn in an interval of 1 hour. The diamonds represent the location and time where/when the injection was observed.

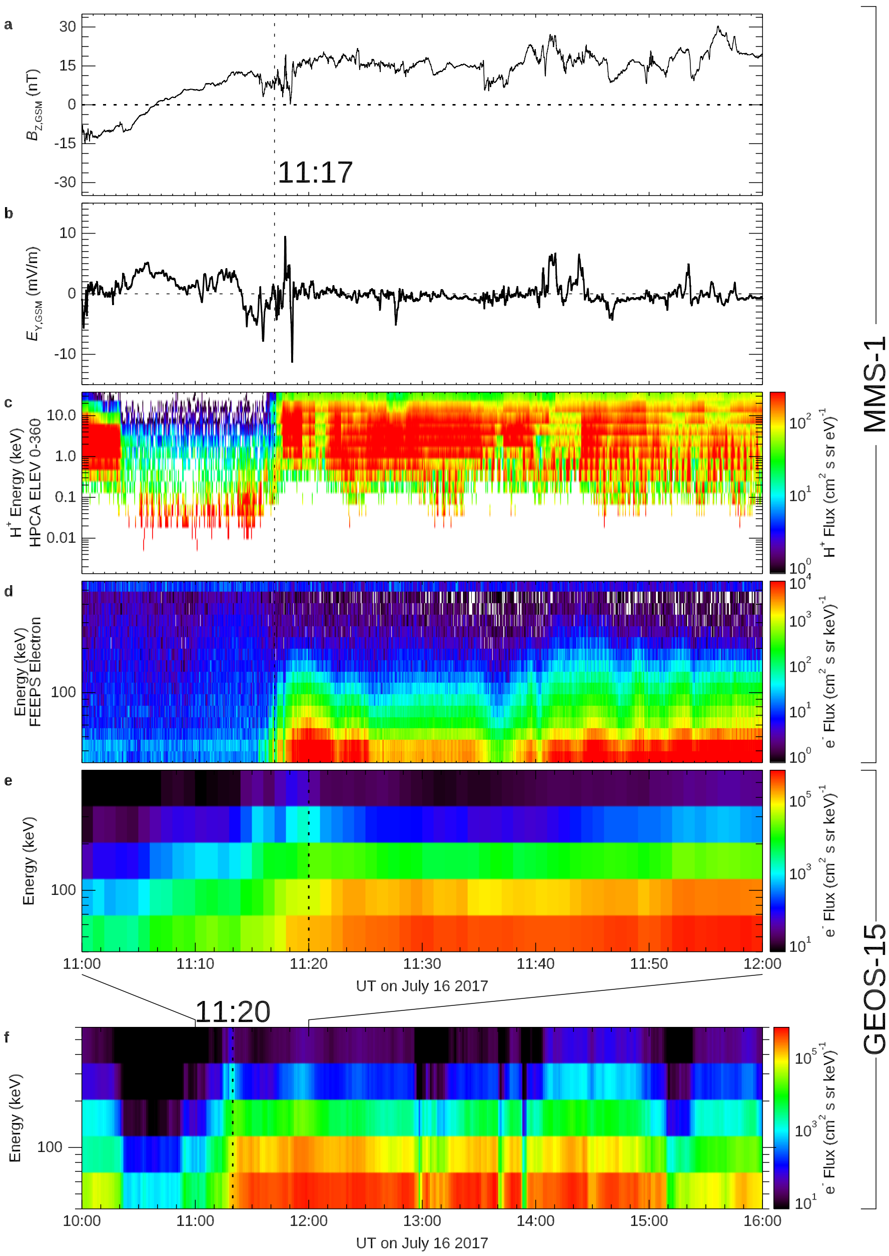

Supplementary Figure 9. Plasma injection observed by MMS-1 and GOES 15 on July 16 2017. From top to bottom shown are MMS-1 observations of **a** z-component of the magnetic field in GSM coordinate system, **b** y-component of the electric field in GSM coordinate system, **c** omni-direction proton differential flux, and **d** omni-direction electron differential flux between 11:00 UT and 12:00 UT. **e** Energetic electron spectrum observed by GOES 15 satellite between 11:00 and 12:00 UT. **f** Energetic electron spectrum observed by GOES 15 satellite between 11:00 and 16:00 UT. The vertical dashed line in **a-d** indicates an injection observed at 11:17 UT. The vertical dashed line in **e-f** indicates the enhancement of electron flux at 11:20 UT. The locations of the satellites are shown in Supplementary Figure 8.

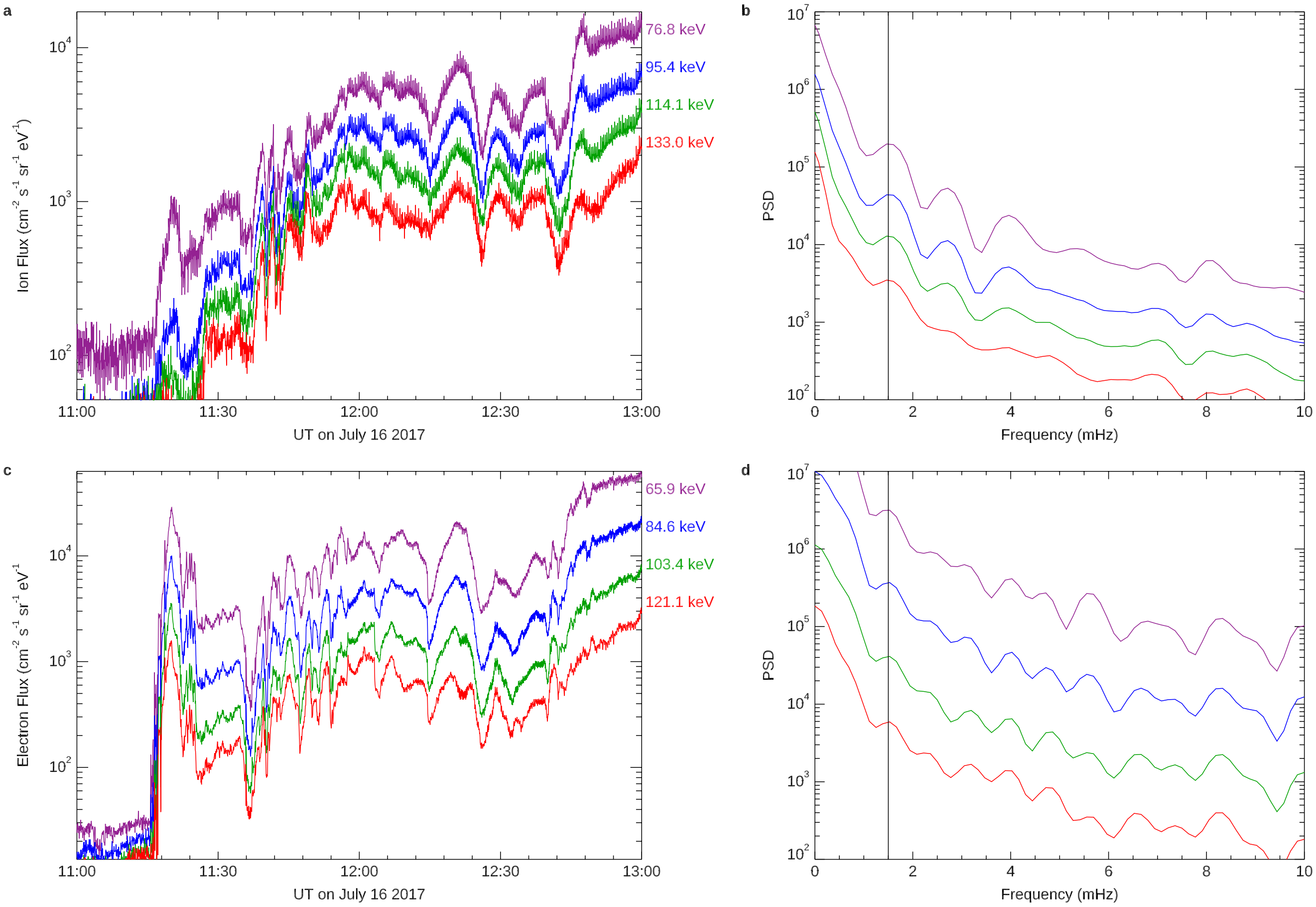

Supplementary Figure 10. Spectral analysis of particle injection observed by MMS-1. a MMS-1 observations of omni-direction ion differential flux at four energy levels. **b** Power spectrum density of the ion flux with the vertical line denoting a frequency of 1.5 mHz. **c** MMS-1 observations of omni-direction electron differential flux at four energy levels. **d** Power spectrum density of the electron flux with the vertical line denoting a frequency of 1.5 mHz.

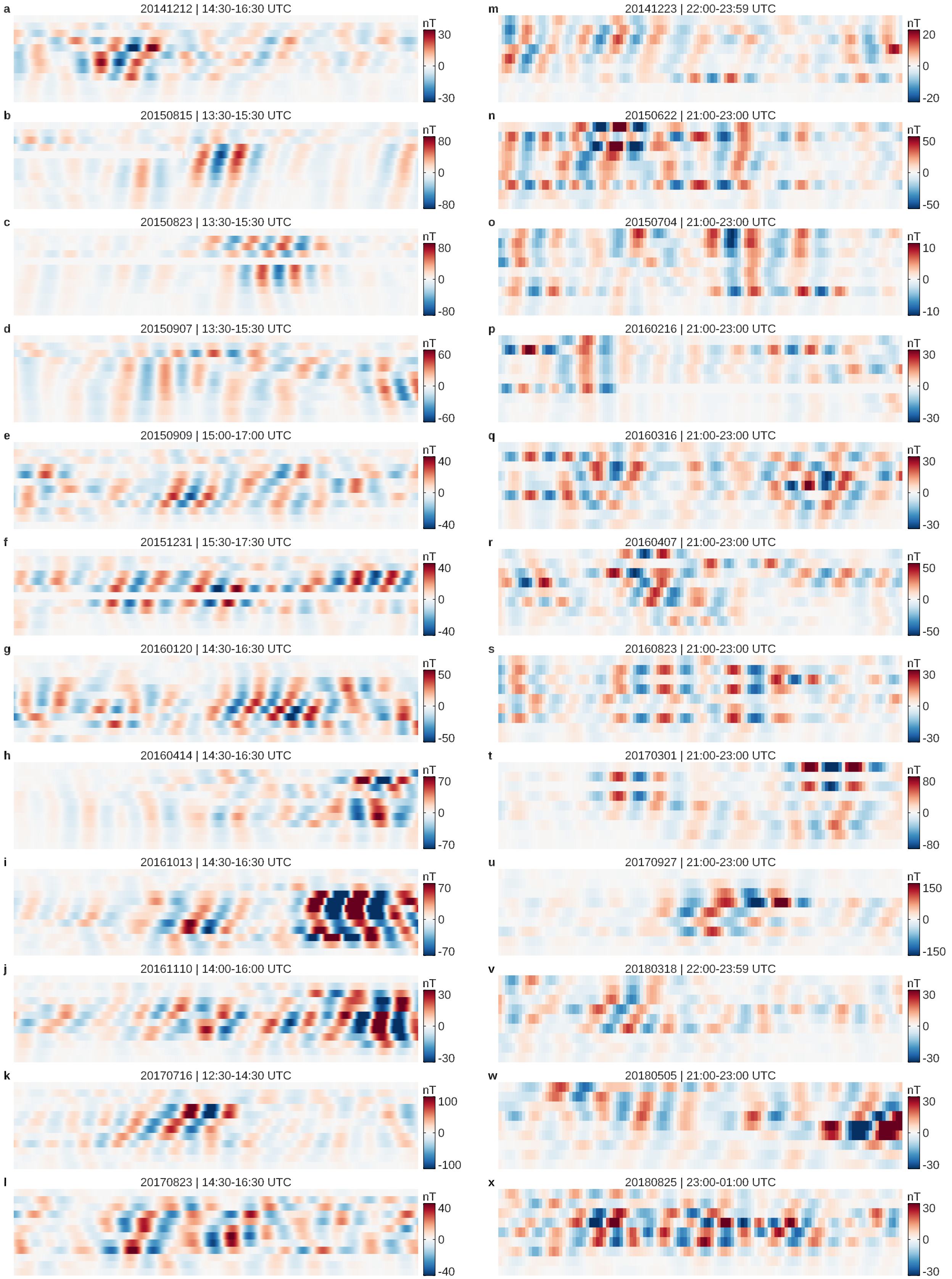

Supplementary Figure 11. ULF (1–2 mHz) waves observed by ground-based station chains. Bandpass filtered (1–2 mHz) perturbations of the N component of the magnetic field observed by the

ground-based station chain in the IMAGE magnetometer array and the SuperMAG database are presented in panels **a-l** and panels **m-x**, respectively. The display formats of each panel are similar to those of Fig. 4c. Each panel presents data in a time range of two hours as indicated at the top of each panel. For the IMAGE chain, the twelve stations correspond to the twelve rows in panels **a-l**. For the SuperMAG database, the nine stations correspond to the nine lines in panels **m-x**. The color bar is shown at the right of each panel.

Supplementary Table 1. List of the geomagnetic storms and SA events.

ID	UTC [a]	Dst (nT)	SA [b]	Satellite	IMF
1	2014-02-19 05:57	-119	Yes	DMSP	South
2	2014-02-20 06:29	-80	Yes	DMSP	South
3	2014-02-23 18:39	-55	Yes	DMSP	South
4	2014-02-27 22:51	-97	Yes	DMSP	South
5	2014-03-13 00:47	-41	Yes	DMSP	South
6	2014-04-11 23:00	-87	Yes	DMSP	South
7	2014-04-30 15:15	-67	Yes	DMSP	South
8	2014-05-04	-47	N/A	DMSP	South
9	2014-05-08 21:04	-46	Yes	DMSP	South
10	2014-08-27 21:18	-79	Yes	DMSP	South
11	2014-09-12 23:19	-88	Yes	DMSP	South
12	2014-10-09 13:33	-50	Yes	DMSP	South
13	2014-10-14 17:35	-49	Yes	DMSP	South
14	2014-10-20 17:22	-49	Yes	DMSP	South
15	2014-10-28	-57	N/A	DMSP	South
16	2014-11-10 15:56	-65	Yes	DMSP	South
17	2014-12-12 13:59	-53	Yes*	DMSP	South
18	2014-12-22 01:32	-71	Yes	DMSP	South
19	2014-12-23 22:01	-57	Yes**	DMSP	South
20	2015-01-04 16:55	-62	Yes	DMSP	South
21	2015-01-07 10:50	-99	Yes	DMSP	South
22	2015-02-18 11:33	-64	Yes	DMSP	South
23	2015-02-24 04:23	-52	Yes	DMSP	South
24	2015-03-01	-46	N/A	DMSP	South
25	2015-03-02 09:14	-55	Yes	DMSP	South
26	2015-03-17 15:52	-222	Yes	DMSP	South
27	2015-04-10 08:49	-51	Yes	DMSP	South
28	2015-04-11 12:05	-73	Yes	DMSP	South
29	2015-04-15 19:18	-71	Yes	DMSP	South
30	2015-05-18 23:58	-44	Yes	DMSP	South
31	2015-06-08 07:34	-71	Yes	DMSP	South
32	2015-06-22 21:27	-121	Yes**	DMSP	South
33	2015-06-23 03:27	-204	Yes	DMSP	South

34	2015-07-04 22:05	-67	Yes**	DMSP	South
35	2015-07-13 10:20	-59	Yes	DMSP	South
36	2015-07-23 06:09	-63	Yes	DMSP	South
37	2015-08-15 12:58	-57	Yes*	DMSP	South
38	2015-08-16 06:46	-84	Yes	DMSP	South
39	2015-08-19 07:01	-50	Yes	DMSP	South
40	2015-08-23 14:34	-43	Yes*	DMSP	South
41	2015-08-27 17:02	-72	Yes	DMSP	South
42	2015-08-28 17:41	-81	Yes	DMSP	South
43	2015-09-04	-44	N/A	DMSP	South
44	2015-09-07 14:38	-70	Yes*	DMSP	South
45	2015-09-09 14:12	-92	Yes*	DMSP	South
46	2015-09-11 08:45	-81	Yes	DMSP	South
47	2015-09-20 10:08	-75	Yes	DMSP	South
48	2015-10-01 19:29	-40	Yes	DMSP	South
49	2015-10-07 05:31	-93	Yes	DMSP	South
50	2015-10-18 09:54	-48	Yes	DMSP	South
51	2015-11-01 12:38	-43	Yes	DMSP	South
52	2015-11-03 17:12	-55	Yes	DMSP	South
53	2015-11-07 06:23	-89	Yes	DMSP	South
54	2015-11-10 10:34	-58	Yes	DMSP	South
55	2015-12-14 18:14	-47	Yes	DMSP	South
56	2015-12-20 07:44	-155	Yes	DMSP	South
57	2015-12-31 13:41	-110	Yes*	DMSP	South
58	2016-01-20 15:09	-93	Yes*	DMSP	South
59	2016-02-01	-48	N/A	DMSP	South
60	2016-02-03	-50	N/A	DMSP	South
61	2016-02-16 20:33	-57	Yes**	DMSP	South
62	2016-03-06 18:26	-98	Yes	DMSP	South
63	2016-03-15 20:41	-49	Yes	DMSP	South
64	2016-03-16 21:16	-56	Yes**	DMSP	South
65	2016-04-03 17:31	-56	Yes	DMSP	South
66	2016-04-07 22:00	-60	Yes**	DMSP	South
67	2016-04-13 00:38	-54	Yes	DMSP	South
68	2016-04-14 14:53	-59	Yes*	DMSP	South
69	2016-04-16 20:01	-55	Yes	DMSP	South
70	2016-05-08 09:08	-88	Yes	DMSP	South
71	2016-08-03 11:22	-52	Yes	DMSP	South
72	2016-08-23 21:14	-74	Yes**	DMSP	South
73	2016-09-01 20:34	-59	Yes	DMSP	South
74	2016-09-28 18:19	-47	Yes	DMSP	South

75	2016-10-13 15:02	-103	Yes*	DMSP	South
76	2016-10-25 09:49	-58	Yes	DMSP	South
77	2016-10-29 08:06	-64	Yes	DMSP	South
78	2016-11-10 15:34	-59	Yes*	DMSP	South
79	2016-11-25 06:13	-46	Yes	DMSP	South
80	2017-03-01 20:55	-61	Yes**	DMSP	South
81	2017-03-27 10:29	-74	Yes	DMSP	South
82	2017-04-22	-46	N/A	DMSP	South
83	2017-05-28 00:48	-125	Yes	DMSP	South
84	2017-07-16 14:30	-65	Yes*	DMSP	South
85	2017-08-23 14:28	-48	Yes*	DMSP	South
86	2017-08-27 20:50	-40	Yes	DMSP	South
87	2017-08-31	-50	N/A	DMSP	South
88	2017-09-08 00:30	-124	Yes	DMSP	South
89	2017-09-27 21:00	-55	Yes**	DMSP	South
90	2017-10-12 00:40	-41	Yes	DMSP	South
91	2017-10-13 17:28	-47	Yes	DMSP	South
92	2017-10-26	-47	N/A	DMSP	South
93	2017-11-07 19:27	-74	Yes	DMSP	South
94	2017-11-21 07:02	-43	Yes	DMSP	South
95	2017-12-04 20:11	-45	Yes	DMSP	South
96	2018-03-18 20:40	-50	Yes**	FY-3D, DMSP	South
97	2018-04-20 10:02	-66	Yes	FY-3D, DMSP	South
98	2018-05-05 22:30	-56	Yes**	FY-3D, DMSP	South
99	2018-06-26 00:50	-48	Yes	FY-3D, DMSP	South
100	2018-08-26 00:33	-174	Yes**	FY-3D, DMSP	South
101	2018-09-10 18:36	-50	Yes	FY-3D, DMSP	South
102	2018-09-22 03:12	-48	Yes	FY-3D, DMSP	South
103	2018-10-07 17:16	-53	Yes	FY-3D, DMSP	South

[a] The total number of the geomagnetic storms with minimum Dst less than -40 nT is 103 during year 2014–2018. SA were observed in 94 out of the 103 storms. For the remaining 9 storms it is not clear whether these auroral structures are present or not. The UTC represents when the SA was most significant.

[b] For this column, 'Yes' means that the SA was observed, 'N/A' means that the observed auroral images were not in the dusk sector or no data was available during the main phase of the storm to determine whether the SA occurred.

* Observations from the ground-based station chain in the IMAGE magnetometer array were available to analysis the ULF geomagnetic pulsations. The stations used are introduced in the Method in the paper. The bandpass filtered N component of the geomagnetic perturbations were presented in Supplementary Figure 11a-l.

** Observations from the ground-based station chain in the North America were available to analysis the ULF geomagnetic pulsations. The stations used are introduced in the Method in the paper. The bandpass filtered N component of the geomagnetic perturbations were presented in the right column of Supplementary Figure 11m-x.
